# NOMPC, a Member of the TRP Channel Family, Localizes to the Tubular Body and Distal Cilium of *Drosophila* Campaniform and Chordotonal Receptor Cells

**DOI:** 10.1002/cm.20493

**Published:** 2010-11-10

**Authors:** Xin Liang, Johnson Madrid, Henri S Saleh, Jonathon Howard

**Affiliations:** Max Planck Institute of Molecular Cell Biology and GeneticsPfotenhauerstrasse 108, Dresden, Germany

**Keywords:** TRPN ion channel, Johnston's organ, mechanotransduction, ciliary dilation, campaniform receptor

## Abstract

Mechanoreception underlies the senses of touch, hearing and balance. An early event in mechanoreception is the opening of ion channels in response to mechanical force impinging on the cell. Here, we report antibody localization of NOMPC, a member of the transient receptor potential (TRP) ion channel family, to the tubular body of campaniform receptors in the halteres and to the distal regions of the cilia of chordotonal neurons in Johnston's organ, the sound-sensing organ of flies. Because NOMPC has been shown to be associated with the mechanotransduction process, our studies suggest that the transduction apparatus in both types of sensory cells is located in regions where a specialized microtubule-based cytoskeleton is in close proximity to an overlying cuticular structure. This localization suggests a transmission route of the mechanical stimulus to the cell. Furthermore, the commonality of NOMPC locations in the two structurally different receptor types suggests a conserved transduction apparatus involving both the intracellular cytoskeleton and the extracellular matrix. © 2010 Wiley-Liss, Inc.

## Introduction

Mechanosensation underlies the perception of sound, touch and acceleration. It is distinguished from other sensory processes by the short, submillisecond, latency between the mechanical stimulus to the cell and the ensuing electrical response [Chalfie,^2009^]. The short latency has led to the notion that forces impinging on the body of an animal are transmitted mechanically to an ion channel, the mechanotransduction channel, whose gating is directly controlled by force. This mechanism is thought to require the physical interaction of multiple elements in a macromolecular transduction apparatus, which includes the channel, the cytoskeleton and extracellular structures [Gillespie and Walker,^2001^]. There are two fundamental questions: what are the molecular components of this apparatus; and how does the proximate stimulus initiate the mechanosensing process? Despite intense study in several model systems, the molecular pathway of mechanotransduction is still not clear.

In the case of hair cells in the vertebrate ear that respond to sounds and accelerations, the mechanotransduction channels have been localized to the distal tips of the stereocilia, actin-filled processes that protrude from the apical surfaces of the cells [Hudspeth,^1989^]. At their distal tips, extracellular connections, termed tip links, connect each stereocilium with its tallest neighbor. It has been proposed that the tip links pull on and gate the channels in response to shearing of the stereocilia by mechanical stimuli [Pickles et al.,^1984^]. The principal components of the tip links have been identified as cadherin 23 and protocadherin 15 [Kazmierczak et al.,^2007^]. However, the identity of the transduction channel, a key component of the transduction apparatus in hair cells, is still a mystery.

In the case of the microtubule-rich touch receptor neurons in *C. elegans*, an essential component of the mechanotransduction channel is MEC-4, a member of the a DEG/ENaC family [Goodman et al.,^2002^; O'Hagan et al.,^2005^; Chalfie,^2009^]. Several other regulatory subunits in the transduction apparatus have also been identified by genetic screens and functional studies, such as MEC-2, MEC-6, MEC-10 [Chalfie and Au,^1989^; Chelur et al.,^2002^; Goodman et al.,^2002^]. It has also been reported that the extracellular matrix component (MEC-1 and MEC-5) and intracellular microtubule (MEC-7 and MEC-12) are all important for the right localization of transduction channel, suggesting an interaction among these structural elements [Emtage et al.,^2004^; Bounoutas et al.,^2009^]. Although the actual proximate stimulus to the channels is still not clear in the *C. elegans* touch response receptor, a recent study on the ultrastructure of these mechanoreceptor has proposed that point compression loads on the cuticle are converted to local membrane bending or stretching by filament-like structures located between the microtubules and plasma membrane [Cueva et al.,^2007^].

In the case of flies, the protein NOMPC in the transient receptor potential N (TRPN) ion channel family has been identified genetically to be necessary for hearing and touch [Walker et al.,^2000^]. The diminished electrical responses of bristle receptors and auditory chordotonal neurons [Kernan et al.,^1994^; Eberl et al.,^2000^] in *nompC* mutant flies suggest that it may be a mechanosensitive channel, though there is no direct evidence that NOMPC is an ion channel. The electrical responses of campaniform receptors, which are closely related to bristle receptors, are also greatly diminished in *nompC* mutant flies (our unpublished observations). If NOMPC is not a transduction channel itself, it is likely to be part of the transduction apparatus because mechanical responses of the hearing organ, thought to be associated with amplification and feedback, are diminished in mutant flies [Gopfert et al.,^2006^]. Orthologs of NOMPC in *C. elegans*, zebrafish and *Xenopus* have also been reported to be involved in mechanosensation [Sidi et al.,^2003^; Shin et al.,^2005^; Li et al.,^2006^; Kang et al.,^2010^]. In *C. elegans* and *Xenopus*, the NOMPC orthologs are localized to cilia [Shin et al.,^2005^; Li et al.,^2006^] suggesting that NOMPC may be an evolutionarily conserved transduction-associated channel of ciliated mechanoreceptors.

Although NOMPC was first identified in flies, its subcellular location has remained undetermined for a long time. This missing information limits the understanding of the working mechanism of fly mechanoreceptors. To localize the protein, we screened a large array of NOMPC protein fragments for their ability to generate monoclonal antibodies in mice. An antibody directed towards the N-terminus of the protein localizes to the distal tips of ciliated mechanoreceptor neurons in both the campaniform sensilla, which detect cuticle deformation, and in chordotonal organs (Johnston's organ and femoral chordotonal organ), which respond to sound and bending of the joints. This subcellular location of NOMPC agrees with two recent reports on the localization of NOMPC in bristle receptors, Johnston's organ and embryonic chordotonal organs by using two different polyclonal antibodies and green fluorescent protein (GFP)-tagged NOMPC [Cheng et al.,^2010^; Lee et al.,^2010^]. The consistent localization of NOMPC in the distal cilia of all fly mechanoreceptors suggests an essential role of NOMPC in mechanosensation, and that mechanical force is transduced at the site where it is transmitted to the mechanoreceptors from adjacent cuticular structures.

## Materials and Methods

### Fly Strains

*nompC^3^*, and *cn bw* flies were the gifts from the lab of Prof. Martin Göpfert (University of Göttingen, Germany). The DsRed-DCX-EMAP fly strain was from Bechstedt et al. [2010].

### Antigen Screening and Antibody Generation

**Fig. 1:**
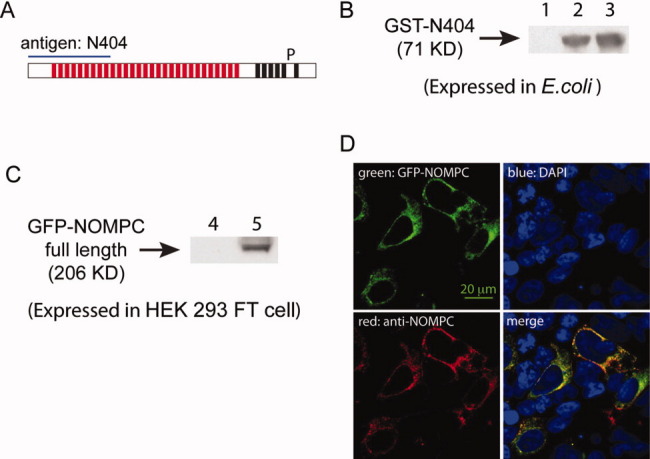
Antigen information and antibody screening **A:** The polypeptide with first 404 amino acids (N404) in the N-terminal of NOMPC was fused with GST and chosen as antigen. **B:** Western blot on the bacterial lysate with anti-NOMPC antibody (clone number: 1214-A02-01). **Lane 1**: uninduced cell; **Lane 2**: induced for 2 hours; **Lane 3**: induced for 4 hours. The band at ∼ 70 KD (GST-N404) was only detected at induced cell sample. **C:** Western blot on cell lysate of HEK 293FT cell. **Lane 4**: wild type HEK 293FT cell; **Lane 5**: HEK 293FT cell transiently transfected with N-terminal GFP-tagged full length NOMPC (NT-GFP-NOMPC). A specific band at ∼ 206 KD was detected only in transfected cell samples. **D:** Immunostaining on the HEK 293FT cells transfected with NT-GFP-NOMPC. Green: NT-GFP-NOMPC channel; Blue: DAPI; Red: anti-NOMPC. Only the cells show the GFP signal (NT-GFP-NOMPC, green) could be stained by anti-NOMPC antibody (red).

**Fig. 2:**
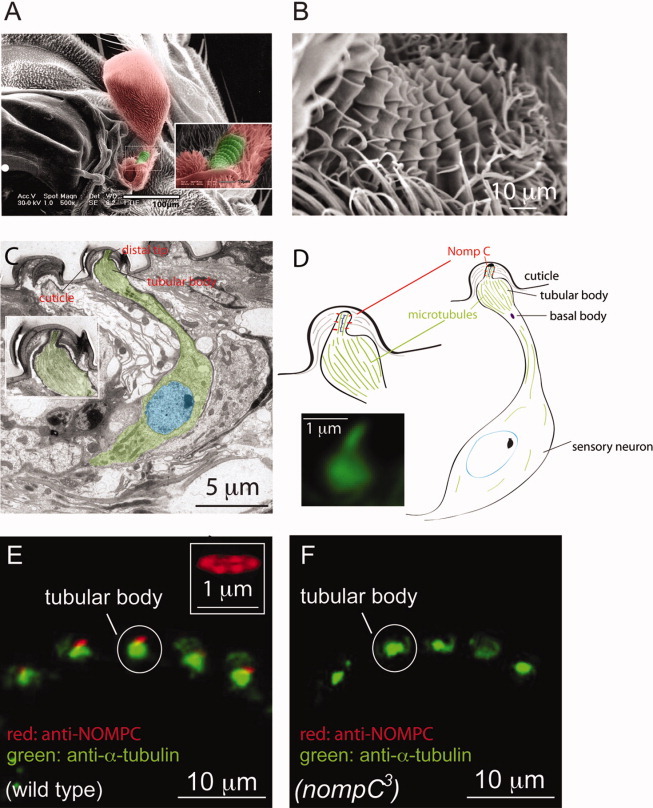
The structure of Campaniform receptor in haltere and the subcellular localization of NOMPC in campaniform receptor in the haltere **A:** SEM image of a haltere and a receptor field (green in inset) in the pedicel of the haltere (pink in inset). Scale bar: 100 μm. **B:** High magnification SEM image of a campaniform receptor field in the haltere pedicel. **C:** Transmission electron microscopy image of a longitude section of a campaniform receptor in the haltere from a *nompC^3^* fly. The whole cell region and nucleus region are highlighted in light green and light blue, respectively. It shows that campaniform receptor in *nompC^3^* fly has a similar overall shape to that of wild type neurons [Keil,^1997^]. The inset shows an enlargement of the tubular body in this campaniform receptor. **D:** Schematic diagram of a campaniform receptor and the tubular body. Inset: the distal tip region of the campaniform receptor is clearly visualized with a mouse anti-acetylated tubulin antibody (Sigma, T6793). **E:** Anti-NOMPC antibody (red) exclusively stains the distal tips of tubular body, here visualized with an anti-α-tubulin antibody (green) (Abcam, Catalog No. ab15246). The inset shows a top-view of anti-NOMPC staining (red) in the distal tip of the tubular body. **F:** The same region of the tubular body (anti-α-tubulin, green) in the *nompC^3^* fly did not stain with anti-NOMPC antibody. The distal tip region is not as strongly stained by the anti-α-tubulin as it is by the anti-acetylated tubulin antibody (D, inset) (Supporting Information Fig. S1).

**Fig. 3:**
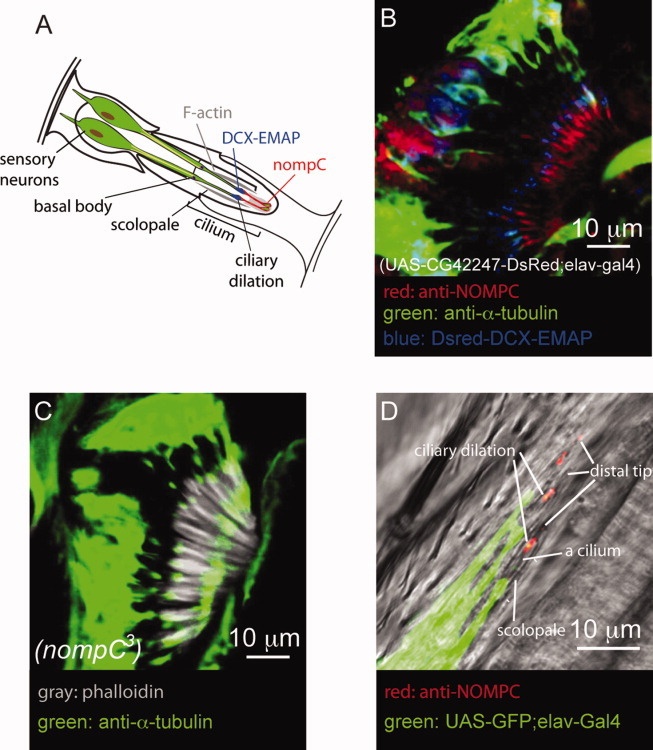
Subcellular localization of NOMPC in Johnston's organ in the antenna and femoral chordotonal organ in the leg **A:** Schematic picture of Johnston's organ cells. **B:** Anti-NOMPC antibody (red) stains the distal cilia of Johnston's organ cells (anti-α-tubulin, green). A fly strain expressing DsRed-tagged DCX-EMAP in the nervous system was used to label the position of ciliary dilation (blue). The strong red staining on the left side of the images is the strong auto-fluorescence from a piece of cuticle in this tissue section. **C:** The distal cilia of Johnston's organ in the *nompC^3^* fly did not stain with anti-NOMPC antibody. To visualize the fine structure of cilia labeled with anti-α-tubulin, anti-α-tubulin signal was over-exposed (green) and phalloidin was also used to label the actin-rich scolopale (gray). **D:** Subcellular localization of NOMPC in leg femoral chordotonal cells. A fly strain expressing GFP in the nervous system was used to label the chordotonal cells. To visualize the fine structure of the cilia, the cytoplasmically expressed GFP signal (green) was over-exposed. We identified the ciliary dilation as the bright dot in the GFP channel (yellow), as expected from the enlargement of the cytoplasm at the dilation; furthermore, the location of the bright dot is approximately two thirds distance from basal body to the distal tip (Supporing Information Fig. S2), the expected location of the dilation based on the detailed ultrastructure [Kernan,^2007^]. Anti-NOMPC antibody (red) stains the distal cilia of femoral chordotonal organ cells, from the dilation to the distal tip, similar to the staining in Johnston's organ.

In total, 36 fragments covering the full length NOMPC cDNA (NOMPC-RA in FlyBase) were cloned into pGEX-6p-2 and expressed in bacteria as glutathione-S-transferase (GST)-tagged polypeptides to screen for their antigenicity and solubility (Protein expression facility, MPI-CBG, Dresden, Germany). The first 404 amino acids (N404) in the N-terminal of NOMPC was chosen as the antigen to immunize mice (Fig. 1A). The mouse monoclonal antibody against NOMPC was produced by fusing B cells isolated from the spleen of a Balb/c mice with the P3x63Ag8.653 myeloma cell line using standard polyethyline glycol (PEG) fusion technology (Antibody facility, MPI-CBG, Dresden, Germany). The positive clones were first screened by ELISA and Western blots using GST-tagged N404 expressed in bacteria (Fig. 1B). Those clones with strongest signal were further tested on human embryonic kidney (HEK) 293FT cells transiently transfected with N-terminal GFP-tagged full length NOMPC (NT-GFP-NOMPC) by immunostaining and Western blot (Figs. 1C and 1D). The NT-GFP-NOMPC was made by cloning full length NOMPC-RA into pcDNA3.1/NT-GFP-TOPO (Invitrogen, Darmstadt, Germany) and transfected into HEK 293FT cells by Fugene HD reagent (Roche, Mannheim, Germany). The number of the clone used for staining in Figs. 2 and 3 is 1214-A02-1.

### Western Blots

For GST-N404 expressed in bacteria, the transformed cells were induced with isopropyl-β-D-thio-galactoside (IPTG) (0.4 mM) for 2 or 4 hours at 37°C with 180 rpm shaking. The cells were homogenized in 1× SDS sampling buffer and loaded onto 3–12% Bis-Tris NuPAGE gel (Invitrogen). For NT-GFP-NOMPC expressed in HEK 293FT cells, the cells were lysed in RIPA buffer (25 mM Tris-HCl (pH 7.6), 150 mM NaCl, 1% NP-40, 1% sodium deoxycholate, 0.1% SDS) on ice for 15 minutes. The cell lysate was clarified by centrifugation at 13,000 rpm for 15 minutes and the supernatant was analyzed by SDS-PAGE. After electrophoresis, the proteins on the gel were transferred to polyvinylidene fluoride (PVDF) membrane. The PVDF membrane was incubated with primary antibody (0.02 μg/ml) at 4°C overnight and then horseradish peroxidase-conjugated secondary antibody (1:500,000) at room temperature for an hour. The signal was detected using ELC advanced detection kit (GE Healthcare, Freiburg, Germany).

### Cryosection Preparation

The haltere and antenna of flies were dissected by sharp forceps and immediately fixed in Stefanini's fixative (1.5 ml picric acid, 2.2 ml formaldehyde, 1.5 ml PIPES stock (0.5 M, pH 7.3) and 4.8 ml H_2_O) by gently shaking for 30 minutes at room temperature. After fixation, the tissue was washed three times with PBS (10 minutes each time) at room temperature. Tissues were then incubated in PBS augmented with 10% sucrose with gentle shaking for 30 minutes and incubated overnight at 4°C in PBS with 25% sucrose. The next day, tissues were embedded in O.C.T. [Richard et al.,^2006^] and frozen quickly in dry ice. The frozen tissue blocks could be stored at −80°C for one month. The frozen blocks were equilibrated at −20°C before sectioning. The thickness of the sections was 5–10 μm (haltere) and 10–15 μm (antenna). The sections were stored at −20°C and used for staining within a week after cutting.

### Immunostaining

For immunostaining of cultured cells, the cells were fixed in 4% paraformaldehyde and permeabilized by PBT (PBS with 0.5% Triton X-100). Then, the fixed cell samples were incubated with primary antibody (20 μg/ml for purified monoclonal antibody against NOMPC) overnight at 4°C. The next day, the sections were washed six times in PBS (5 minutes each time) and then incubated in appropriate Alexa-conjugated secondary antibody (1:200 dilution, Invitrogen) overnight at 4°C. On the third day, the cell samples were washed six times in PBS (5 minutes each time) and imaged by confocal microscopy (Zeiss LSM 510). For immunostaining of tissue sections, the sections stored at −20°C were placed at room temperature for about 5 minutes and washed three times (15 minutes each time) in PBT. For whole-mount tissue staining, the tissues are freshly collected in PBT and then fixed at room temperature for 1 hour in Stefanini's fixative or 4% paraformaldehyde augmented with 0.5% Triton X-100. In the next step, the antibody staining protocols for tissue sections and whole-mount tissues were the same as those for cell samples described above. After the last washing step, the tissue sections and tissues were mounted in Mowiol medium for confocal microscopy.

### Scanning Electron Microscopy

Flies between 1 and 3 days post eclosion were anesthetized with CO_2_. Their halteres were dissected or whole flies were fixed in 2% glutaraldehyde in 0.1 M phosphate buffer at pH 7.2 for 2 hours. Samples were subjected to sequential dehydration with 70, 80, 90, 96 and 100% ethanol. Following dehydration with 100% acetone, the samples were mounted on a stub using carbon tape and sputter-coated with gold. Figure 2A was imaged by scanning electron microscopy (SEM; XL 30 ESEM FEG, FEI-Philips, Kassel, Germany) at 30 kV and Fig. 2B was imaged by SEM (JSM-7500F, JEOL, Eching b. München, Germany) using a back scattered electron detector at an acceleration voltage of 5 kV.

### Transmission Electron Microscopy

Flies between 1 and 3 days post eclosion were anesthetized with CO_2_. Their halteres were dissected and fixed in 2% glutaraldehyde in 0.1 M phosphate buffer at pH 7.2 for 2 hours and post fixed with 1% OsO_4_. The samples were sequentially dehydrated using gradient series of 70, 80, 90, 96 and 100% ethanol, followed by final dehydration step in 100% acetone. The samples were then subjected to infiltration in epon-acetone series of 1:2 and 2:1 mix for 1 hour each, followed by 100% epon series overnight. Then the samples were embedded in epon. Serial, ultrathin sections (50 nm) were cut with a diamond knife on a Leica Ultracut S microtome and collected on Formvar-coated copper slot grids, post stained with 2% uranyl acetate in 70% methanol followed by 0.4% Reynold's lead citrate and imaged in a TECNAI 12 transmission electron microscope (FEI, Eindhoven, Netherlands) operated at 100 kV.

## Results

We localized NOMPC in the campaniform sensilla in *Drosophila*. Campaniform sensilla are related to bristle-type sensilla and sense deformations of the cuticle during walking, jumping and flying. Campaniform receptors are mainly found in the legs, wings and halteres. The halteres, the degenerate rear wings that serve as gyroscopes, have hundreds of campaniform receptors distributed in five fields at the haltere's base (Figs. 2A and 2B). The receptors provide feedback information during flight. The sensory receptor cells of the campaniform sensilla contain a modified cilium whose distal end, termed the tubular body, is closely associated with the cuticle (Fig. 2C). Electron microscopy images show that the tubular body comprises two distinct parts [Keil,^1997^]: a larger proximal part and a smaller distal tip (Figs. 2C and 2D). The distal tip region, which is visualized clearly using an antibody against acetylated tubulin (Fig. 2D, inset), contains a highly ordered array of microtubules embedded in an electron-dense material [Bechstedt et al.,^2010^] and is enclosed by an elaborated extracellular structure that connects to the overlying cuticle (Fig. 2C).

To localize NOMPC, we generated a monoclonal antibody against the first 9-ankyrin-repeats in the N-terminal of NOMPC (see Methods and Fig. 1). The antibody exclusively stained the distal tips of the tubular bodies in campaniform neurons of the halteres (red, Fig. 2E, side view of tubular body). This is consistent with the distal cilia localization of NOMPC in bristle mechanoreceptors [Lee et al.,^2010^] as expected from the close relationship between campaniform and bristle sensilla. Higher magnification images showed that the anti-NOMPC antibody staining pattern was oval-shaped (top view of campaniform receptor), with higher intensity in the periphery than in the middle (inset in Fig. 2E); this is consistent with the shape of the distal tip seen by electron microscopy [Keil,^1997^] and the expected membrane association of the NOMPC in the tip region. The staining was absent in *nompC* null mutants (*nompC^3^*, Fig. 2F), confirming the specificity of our antibody.

We also localized NOMPC in chordotonal organs. Chordotonal organs sense rotation of the joints between different body parts, for example during locomotion. In addition, flies contain a large chordotonal organ, called Johnston's organ, located in the second antennal segment, which senses sound, wind and gravity [Kamikouchi et al.,^2009^; Yorozu et al.,^2009^]. The dendrite of the sensory receptor in Johnston's organ projects from the cell body to the overlying cuticular scolopale cap and contains a nonmotile “9 + 0” cilium. The cilium has a dilation, containing an electron-dense material, about two thirds of the way to the distal end (Fig. 3A).

The anti-NOMPC antibody stained the distal regions of the cilia (red, Fig. 3B). The staining extended from the ciliary dilation, marked by DsRed-DCX-EMAP [Bechstedt et al.,^2010^] (blue, Fig. 3B), to the distal tip of the cilium, marked by actin in the adjacent scolopale cells (actin channel not shown in this panel, but see Fig. 3C). A similar staining pattern to that in Johnston's organ was found in the large femoral chordotonal organs in the joints of the legs (Fig. 3D), which also agrees with the NOMPC localization found in embryonic chordotonal organs [Cheng et al.,^2010^]. The antibody staining region is the site of close apposition of the cilium to the overlying cap cell (Fig. 3C). This staining signal, like the staining in the campaniform receptors, is also absent in the *nompC* null mutant (*nompC^3^* in Fig. 3C).

## Discussion

Our data show that NOMPC localizes to the distal cilium in both campaniform and chordotonal mechanoreceptors. Because of the association of NOMPC with mechanotransduction (see Introduction), our results provide new insight into the transduction mechanism in flies.

First, the distal localization of NOMPC suggests that the site of transduction is where the extracellular structures make physical contact with the neuron. In the case of the campaniform receptor, deformation of the cuticle squeezes the distal tip of the tubular body (Figs. 2C and 2D) [Keil,^1997^]. In the case of the chordotonal receptor, joint rotation stretches the cilium due to its distal attachment to the cap. Localization of the transduction apparatus at the contact sites argues against a mechanism in which the mechanical signal is transmitted intracellularly to a distant site, such as the base of the cilium.

Second, the localization suggests that the prominent microtubule-based cytoskeletal plays a crucial role in transduction. The most likely role is that the microtubules act as a rigid structure against which compressive or tensile forces exerted through the extracellular cuticle can squeeze or stretch the channel and lead to gating. Because of the high rigidity of both the microtubules and the cuticle, it is likely that the transduction apparatus contains a compliant element to protect the channel from excessive forces. The 29 ankyrin repeats in the N-terminus of the NOMPC protein may serve as such an element, acting as a “gating spring” to transmit forces arising from cuticle deformations to the gate of the channel [Howard and Bechstedt,^2004^].

Third, the presence of NOMPC, the microtubule-based cytoskeleton and the extracellular matrix in the same local region in both cell types, suggest that both receptor types may share a conserved mechanotransduction apparatus that senses force transmitted through a cuticular capping structure. Such conservation is interesting because there are striking structural and mechanical differences between the two cell types. The “9 + 0” cilium of chordotonal receptors has C_9_ rotational symmetry, whereas the elliptical distal tip of the tubular body of campaniform receptors has approximate D_2_ dihedral symmetry. Furthermore, the chordotonal receptor is excited by longitudinal tension of the cilium, whereas the campaniform receptor is thought to be excited by transverse compression of the tubular body, as in the case of the bristle receptor [Keil,^1997^]. Though longitudinal tension is likely to lead to transverse compression of the ciliary dilation, it is not clear how a similar proximate mechanical stimulus could lead to channel gating in both cell types. These differences highlight our uncertainty about the precise roles of NOMPC in these two receptor types.

Fourth, the localization of NOMPC suggests that mechanosensory information travels anterogradely from the distal tip to the proximal base of the mechanosensory dendrite. In the proximal part of the chordotonal cilium, below the dilation, two transient receptor potential V (TRPV) ion channels (Inactive and Nanchung) form a complex that is essential for hearing [Kim et al.,^2003^; Gong et al.,^2004^]. Though mutants of *Inactive* and *Nanchung* have no electrical response to sound, they do have active mechanical responses [Gopfert et al.,^2006^] indicating that the mechanotransduction apparatus is likely to be still present in these mutants. By contrast, electrical responses are attenuated and mechanical responses are diminished in NOMPC mutants [Gopfert et al.,^2006^] consistent with NOMPC being a component of the mechanotransduction apparatus, as mentioned in the Introduction. Mechanical measurements indicate that the TRPV channels are downstream of the mechanotransducer [Gopfert et al.,^2006^]. These observations, together with our NOMPC localization suggest the following model: the electrical signal is initiated in the distal region of the mechanoreceptors by a transduction complex that includes the NOMPC protein, and TRPV channels shape the electrical signal as it is transmitted down the cilium to the cell body, where it initiates action potentials that convey the sensory information to the central nervous system.
