# Supplementary material for: NOMPC, a Member of the TRP Channel Family, Localizes to the Tubular Body and Distal Cilium of Drosophila Campaniform and Chordotonal Receptor Cells
Source: Cytoskeleton (Hoboken). 2010 Nov 10;68(1):1–7. doi: 10.1002/cm.20493 (PMC3048163; doi:10.1002/cm.20493)
Supplement: Supplementary file 1 [file cm0068-0001-SD1.pdf]

NOMPC, a member of the TRP Channel Family, Localizes to the Tubular Body and Distal Cilium of *Drosophila* Campaniform and Chordotonal Receptor Cells.

Xin Liang[1], Johnson Madrid[1], Henri S. Saleh[1] and Jonathon Howard[1,*]

Supplementary Information

Figure S1. Different anti-tubulin antibodies give different staining on the distal tip of campaniform receptor.

Figure S2. NOMPC staining in femoral chordotonal organ.

Supplementary Information

Figure S1.

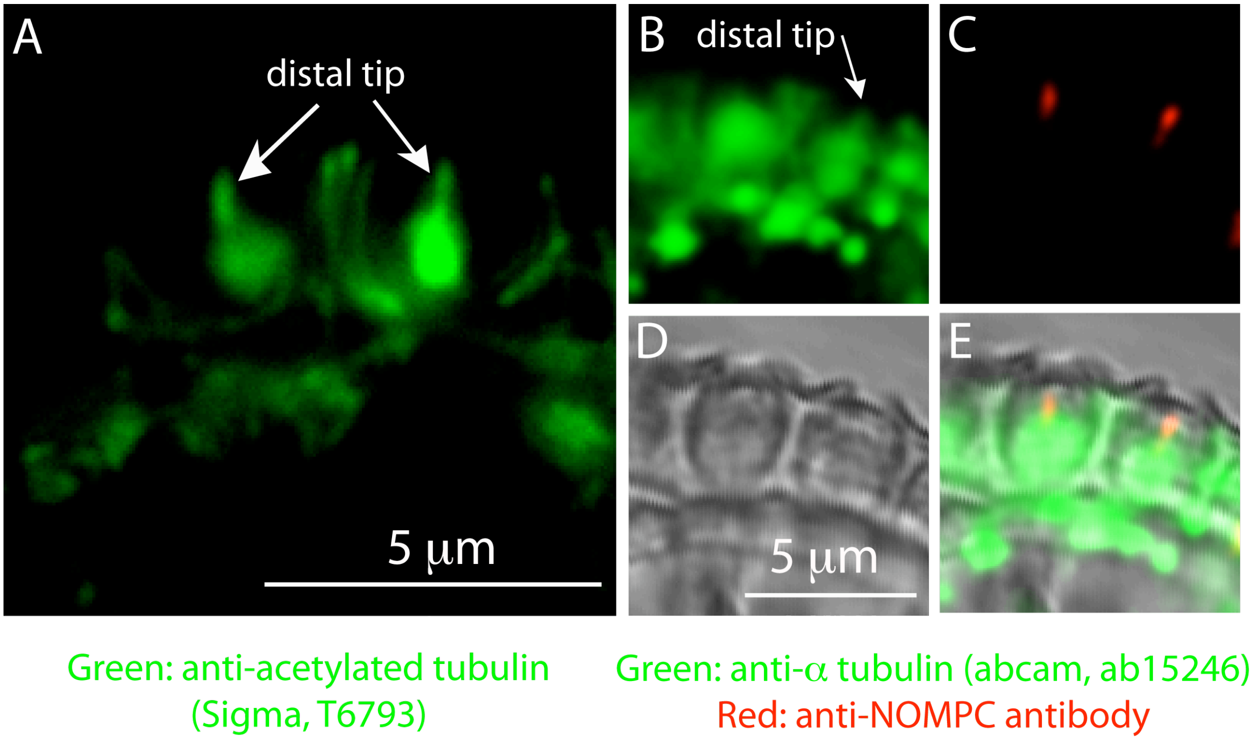

Figure S1. Different anti-tubulin antibodies give different staining in the distal tip of campaniform receptor. **A** Campaniform receptor stained with mouse monoclonal anti-acetylated tubulin antibody (Sigma, T6793). The distal tip region is clearly visible (white arrow). **B** Campaniform receptor stained with rabbit polyclonal anti-alpha tubulin antibody (abcam, ab15246). The distal tip region is not as clearly visible (arrow). **C** Campaniform receptor stained with anti-NOMPC antibody (red). **D** DIC image of campaniform receptor. **E** The composite image of B,C,D.

Figure S2.

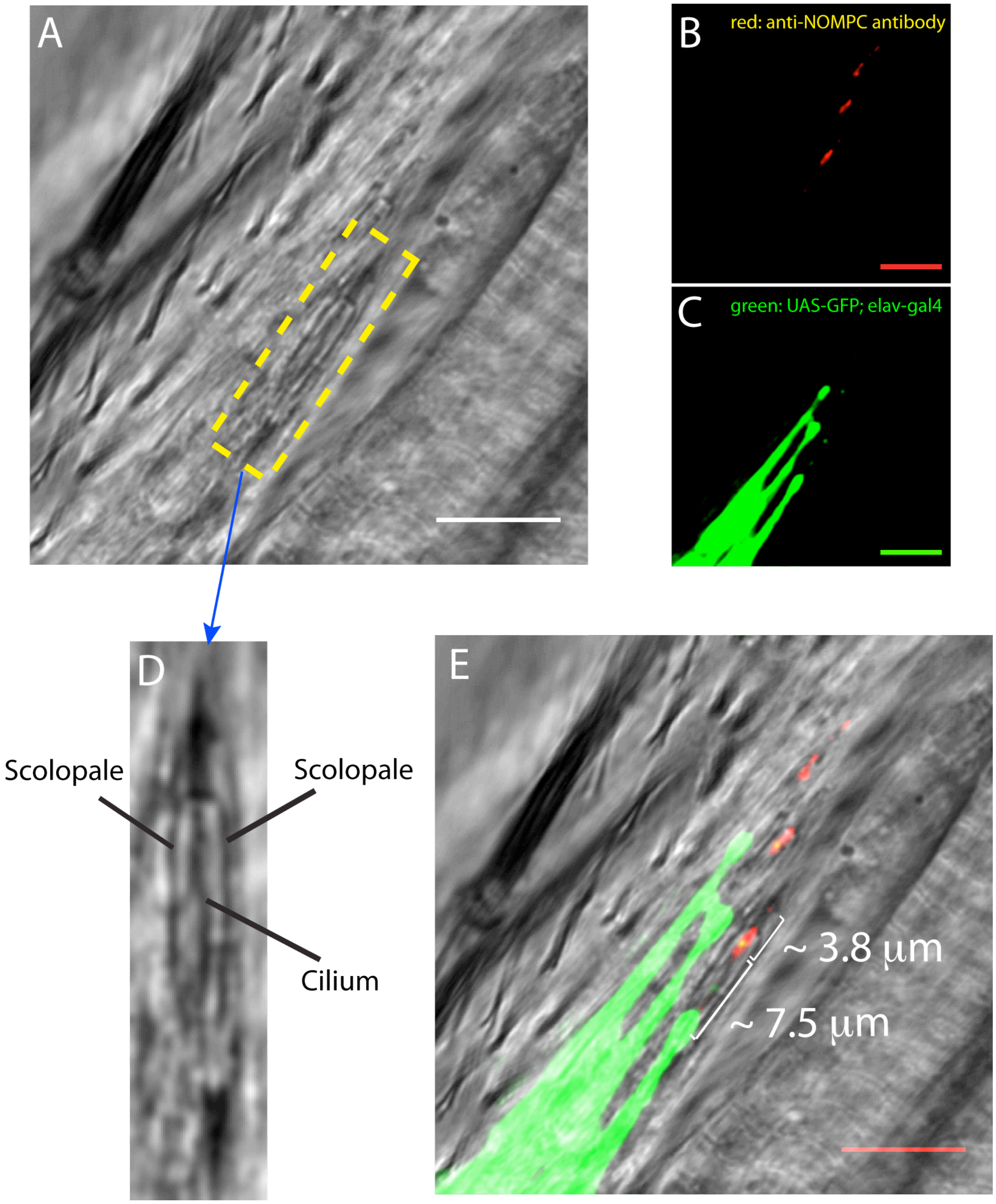

Figure S2. NOMPC staining in the femoral chordotonal organ. **A** The DIC channel image of a chordotonal organ cell. The cilial region is highlighted by the yellow dashed-line box. **B** The NOMPC fluorescence channel of the region shown in **A**. **C** The GFP signal channel. **D** The enlarged picture of the highlighted region in **A**, the scolopale structure and the cilium is clear. **E** The ciliary dilation is identified as the bright dot (yellow) in the GFP channel due to the enlargement of the cytoplasm in this region. The distance measurement shows that the bright dot is about two thirds of the way from the

basal body to the distal end of cilia, as expected for the ciliary dilation from the detailed ultrastructure **(Kernan 2007)**. Scale bar: 10 μm.

Reference

Kernan MJ. 2007. Mechanotransduction and auditory transduction in Drosophila. Pflugers Arch 454(5):703-20.
